# Effect of Curcumin on Protein Damage Induced by Rotenone in Dopaminergic PC12 Cells

**DOI:** 10.3390/ijms21082761

**Published:** 2020-04-16

**Authors:** Sandra Buratta, Elisabetta Chiaradia, Alessia Tognoloni, Angela Gambelunghe, Consuelo Meschini, Luigi Palmieri, Giacomo Muzi, Lorena Urbanelli, Carla Emiliani, Brunella Tancini

**Affiliations:** 1 Department of Chemistry, Biology and Biotechnology, University of Perugia, 06123 Perugia, Italy; sandra.buratta@unipg.it (S.B.); consuelo.meschini@studenti.unipg.it (C.M.); luigi.palmieri@studenti.unipg.it (L.P.); lorena.urbanelli@unipg.it (L.U.); carla.emiliani@unipg.it (C.E.); 2 Department of Veterinary Medicine, University of Perugia, 06126 Perugia, Italy; elisabetta.chiaradia@unipg.it (E.C.); alessia.tognoloni@studenti.unipg.it (A.T.); 3 Department of Medicine, University of Perugia, 06132 Perugia, Italy; angela.gambelunghe@unipg.it (A.G.); giacomo.muzi@unipg.it (G.M.)

**Keywords:** rotenone, curcumin, PC12 cells, protein oxidation, oxidative stress, Parkinson’s disease

## Abstract

Oxidative stress is considered to be a key factor of the pathogenesis of Parkinson’s disease, a multifactorial neurodegenerative disorder characterized by reduced dopaminergic neurons in the *substantia nigra pars compacta* and accumulated protein aggregates. Rotenone is a worldwide-used pesticide that induces the most common features of Parkinson’s by direct inhibition of the mitochondrial complex I. Rotenone-induced Parkinson’s models, as well as brain tissues from Parkinson’s patients, are characterized by the presence of both lipid peroxidation and protein oxidation markers resulting from the increased level of free radical species. Oxidation introduces several modifications in protein structure, including carbonylation and nitrotyrosine formation, which severely compromise cell function. Due to the link existing between oxidative stress and Parkinson’s disease, antioxidant molecules could represent possible therapeutic tools for this disease. In this study, we evaluated the effect of curcumin, a natural compound known for its antioxidant properties, in dopaminergic PC12 cells treated with rotenone, a cell model of Parkinsonism. Our results demonstrate that the treatment of PC12 cells with rotenone causes severe protein damage, with formation of both carbonylated and nitrotyrosine-derived proteins, whereas curcumin (10 µM) co-exposure exerts protective effects by reducing the levels of oxidized proteins. Curcumin also promotes proteasome activation, abolishing the inhibitory effect exerted by rotenone on this degradative system.

## 1. Introduction

Oxidative stress is strongly implicated in the pathogenesis and pathophysiology of many diseases, including cancer, diabetes and neurodegenerative disorders [1,2]. The increase in free radicals (FR) such as reactive oxygen species (ROS) or nitrogen reactive species (RNS) causes DNA damage, lipid peroxidation and protein oxidation [3,4]. The brain, in particular, is very susceptible to FR exposure. Indeed, oxidative stress and mitochondrial dysfunction play a crucial role in the pathogenesis of Parkinson’s disease (PD) [5,6,7], a multifactorial neurodegenerative disorder characterized by the progressive loss of dopaminergic neurons in the *substantia nigra pars compacta* and by the presence of abnormal protein aggregates known as Lewy bodies [6]. In addition to misfolded and aggregated α-synuclein, other proteins, such as components of the ubiquitin proteasome system (UPS) and chaperones, have also been detected in Lewy bodies [8,9], indicating the unsuccessful attempt of UPS to remove the proteinaceous aggregates. Many recent reports indicated altered protein quality control and UPS impairment as being among the main causative factors involved in many neurodegenerative disorders, including PD [10,11].

Although the pathogenic molecular mechanism of PD has not been fully elucidated, it is widely recognized that a combination of genetic and environmental factors significantly contributes to the neurodegenerative process responsible for the dopaminergic neuron death. Cumulating evidence suggests that pesticide exposure may be one of the major environmental risk factors contributing to the PD pathogenesis [12]. Several pesticides, such as rotenone (RT), paraquat, dieldrin and maneb, have been used to develop animal/cell Parkinsonian models, which recapitulate the dopaminergic neuron degeneration and the most common cellular hallmarks of the disease, such as oxidative stress, mitochondrial dysfunction and impaired proteasomal function [13,14]. RT is a worldwide-used pesticide which induces specific inhibition of the mitochondrial complex I (NADH-dehydrogenase) and, in turn, mitochondrial metabolism alterations, ATP level decrease and ROS level raise [12]. Moreover, it has been reported that RT exposure leads to the inhibition of the proteasomal activity [12]. Analogously to brain tissues from PD patients, in vivo and in vitro RT-induced PD models are characterized by the presence of both lipid peroxidation and protein oxidation markers resulting from oxidative stress damage [15,16,17,18]. Protein oxidation leads to the formation of protein carbonyls, sulfoxides and nitrotyrosines, which compromise their biological function. In particular, carbonylated proteins are commonly used as markers of oxidative stress in cells and tissues, due to their stability and early formation [4,19]. In order to get more insight into the mechanism linking oxidative protein damage and cellular dysfunction, in a previous work, we analyzed carbonylated proteome in dopaminergic PC12 cells treated with RT [20], a cellular model of Parkinsonism. This study identified many carbonylated proteins which are involved in pivotal cellular processes that are altered in PD, including energy metabolism, neurotransmitter biosynthesis and protein homeostasis system.

The link between oxidative stress and PD suggests a possible protective and therapeutic role of antioxidants [1,21]. Among these, the natural polyphenol curcumin (CURC) received special attention not only for its antioxidant properties, but also for its anti-inflammatory, anticancer and neuroprotective activities [22,23]. CURC is the major active component extracted from the rhizome of *Curcuma longa*, and it has been known since antiquity for its numerous medicinal effects [23]. It has been suggested as a promising compound for phytomedicine. In particular, CURC demonstrated antioxidant properties in various cell and animal models of neurodegenerative disorders [24]. Several studies showed that CURC has a protective effect against oxidative-stress-inducing antioxidant response [25]. Notably, evidence has also been produced that supports the efficacy of CURC in PD. In both cellular and animal models of PD, CURC protects from oxidative stress by reducing the production of ROS and malondialdehyde and restoring GSH levels [26,27,28,29]. More specifically, antioxidative and antiapoptotic activity of CURC has been reported in RT-treated PC12 cells, thus suggesting neuroprotective effect of this compound on dopaminergic neurons [30]. In our previous study carried out on PC12 cells, we described RT-induced carbonylation of proteins involved in protein quality control systems, including molecular chaperones, UPS components and autophagy-lysosome pathway members [20]. Oxidative modifications of these proteins are consistent with the failure of the protein processing systems in preventing the cytotoxic protein aggregation and removing the misfolded protein inclusions observed in PD.

In order to get more insight on the molecular mechanism underlining the protective effect of CURC against oxidative stress in PD models, in this study, we investigated the effect of CURC on accumulation of oxidatively modified proteins in dopaminergic PC12 cells treated with RT and evaluated its efficacy to rescue the functionality of proteasomal degradation system.

## 2. Results

It is well-known that CURC exerts many biological effects, and some of them could be explained through its antioxidant properties. In this study, we carried out experiments to evaluate whether CURC exerts protective effects against RT-induced oxidative damage in PC12 cells, focusing on oxidized protein levels, a parameter which can contribute to the protein aggregation, one of the main features of PD. In addition, the effect of RT and CURC on proteasome activity was also investigated.

**Figure 1:**
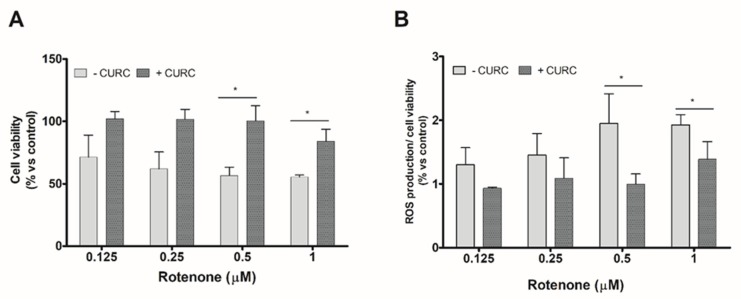
Protective effect of curcumin on cell viability and reactive oxygen species (ROS) production induced by rotenone in PC12 cells. Cells were treated with RT at the indicated concentrations, for 24 h, in the absence (−) or presence (+) of CURC (10 μM). (A) Cell viability by 3-(4,5-di- methylthiazol-2-yl) -2,5-diphenyltetrazolium bromide (MTT) assay. Results are expressed as percentages with respect to controls (cells incubated in the absence of RT). (B) Intracellular ROS production assessed by 2′,7′-dichlorodihydro fluorescein diacetate (DCFH-DA) probe. Normalized DCF fluorescence values were expressed as percentages with respect to controls (cells incubated in the absence of RT). (* *p* < 0.05 CURC-treated cells vs. cells incubated in absence of CURC.)

We evaluate, firstly, the viability of PC12 cells incubated with various concentrations of RT in the absence or presence of CURC. The concentration of CURC (10 µM) used in these experiments has been described as being nontoxic for cell viability [30]. Anyway, viability of PC12 cells incubated with various concentrations of CURC (1–10 µM) was evaluated; none of the tested CURC concentrations induced cytotoxic effects. The results reported in Figure 1 confirmed that RT is able to reduce PC12 cell viability and induces oxidative stress and showed that the higher effects are observed at the highest concentrations tested (0.5 and 1 µM). The co-incubation of RT with CURC (10 µM) reduced the cytotoxic effects induced by RT (Figure 1A). Similar experiments were performed to assess the ability of CURC to reduce the RT-induced intracellular ROS level increase. As shown in Figure 1B, CURC was able to reduce the production of ROS induced by the highest concentrations of RT.

Then, we evaluated the protective effect of CURC on protein oxidation induced by RT. In particular, levels of carbonylated proteins, which are early markers for protein oxidation, and nitrotyrosine-modified proteins, which are indicative of nitrosative stress, were assessed in PC12 cells incubated with RT in the absence or presence of CURC.

**Figure 2:**
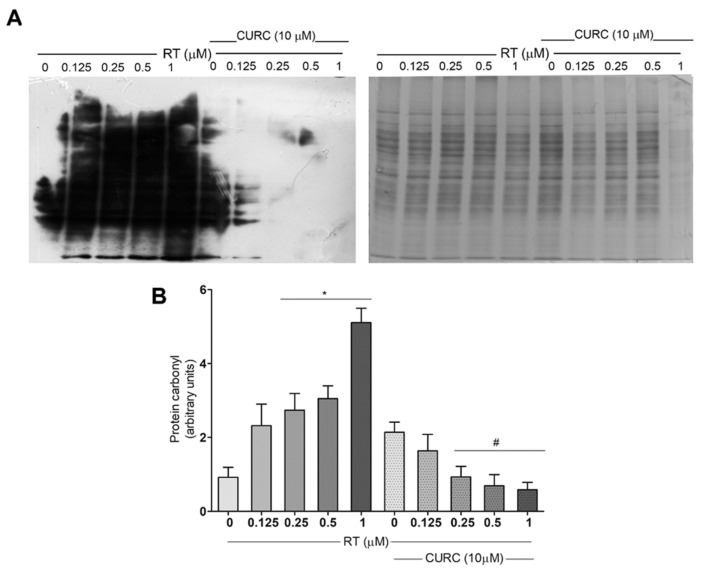
Protective effect of curcumin on rotenone-induced protein carbonylation in PC12 cells. Cells were treated with RT at the indicated concentrations, for 24 h, in the absence or presence of CURC (10 µM). (A) Representative immunoblot of carbonylated proteins, using an antibody against DNP and the corresponding Coomassie-stained polyvinylidene difluoride (PVDF) membranes. (B) Bar graph of carbonylated protein levels obtained by densitometric analysis. Results are reported as ratio between the optical density (OD) obtained from the whole lane on film and the OD of the corresponding lane in Coomassie-stained PVDF membrane. (* *p* < 0.05 vs. control cells (0 RT), ^#^ *p* < 0.05 vs. the exposed to 0.25, 0.5, 1 μM RT alone.)

Protein carbonylation levels were assessed after the conjugation of carbonyl residues with 2,4-dinitrophenylhydrazine (DNPH), followed by immunoblotting, using an antibody against DNP. Figure 2 shows carbonylated protein profiles in PC12 cells treated with various concentrations of RT (0.1–1 µM), in the absence or presence of CURC (10 µM). Results here reported, in addition to confirming that RT induced a dose-dependent increase in protein carbonylation levels in PC12 cells [20], demonstrate that the presence of CURC during RT treatment significantly reduced the formation of carbonylated proteins (Figure 2). No significant differences were found in the levels of carbonylated proteins in cells incubated with CURC alone or in co-presence with RT, with respect to control cells. These results highlighted the protective effects of CURC on RT-induced protein carbonylation.

**Figure 3:**
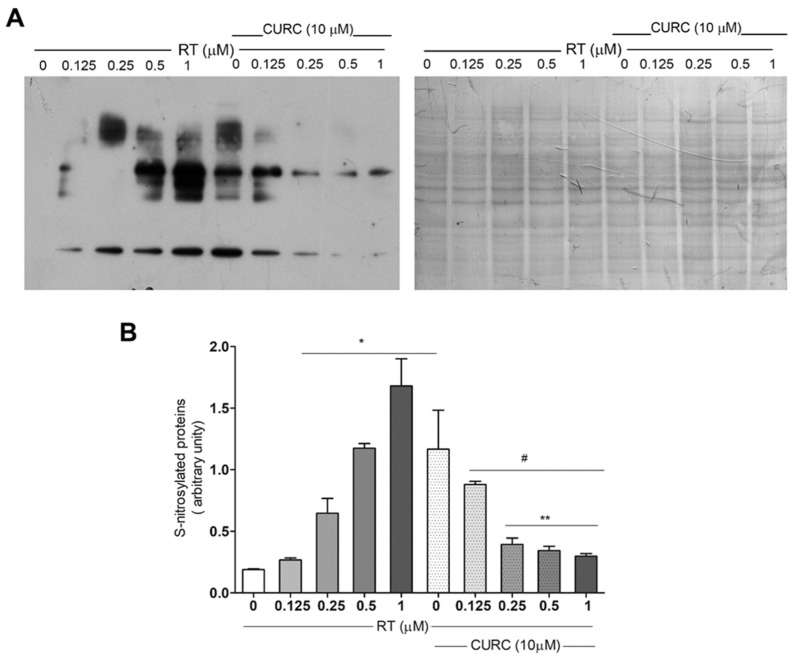
Protective effect exerted by curcumin on rotenone-induced nitrotyrosine-modified proteins in PC12 cell. Cells were treated with RT at the indicated concentrations, for 24 h, in the absence or presence of CURC (10 µM). (A) Representative immunoblot of nitrated proteins, using an anti-nitrotyrosine antibody and the corresponding Coomassie-stained PVDF membrane. (B) Bar graph of nitrotyrosine-modified protein levels analyzed by densitometric analysis. Results are reported as ratio between the optical density (OD) obtained from the whole lane on film and the OD of the corresponding lane in Coomassie-stained PVDF membrane. (* *p* < 0.05 vs. cell controls (0 RT), ^#^ *p* < 0.05 vs. cells exposed to 1 μM RT, ** *p* < 0.05 vs. cells exposed to 0.5 μM RT.)

Although data indicating the increase of protein carbonylation in the PD model consisting of RT-treated PC12 cells [20] have already been published, no literature data exist about oxidative modification of proteins related to nitrotyrosine formation in this cell line treated with RT. Thus, we evaluated protein tyrosine nitration, by immunoblotting, using an antibody against nitrotyrosine in PC12 cells treated with RT, in the absence or presence of CURC (Figure 3). Immunoblotting analyses showed a significant dose-dependent increase in protein nitration upon RT cell treatment (Figure 3B). CURC significantly reduced the levels of immunoreactive proteins when present in culture medium with RT. However, in cells exposed to CURC alone, the levels of nitrotyrosine-modified proteins are higher than controls (Figure 3B).

**Figure 4:**
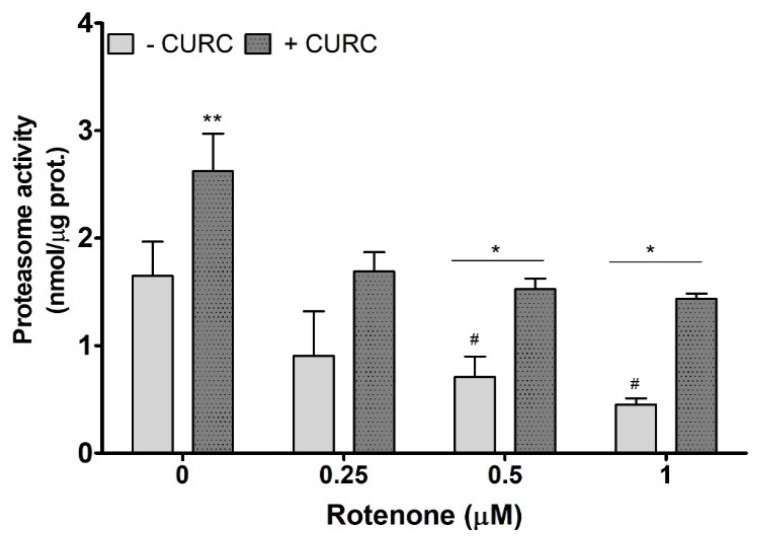
Proteasome activity in PC12 cells incubated with rotenone in the absence or presence of curcumin. Cells were treated with RT at the indicated concentrations, for 24 h, in the absence (-) or presence (+) of CURC (10 µM). At the end of incubation, cells were lysated, and proteasome activity was assessed fluorometrically, using a commercial proteasome activity assay kit (see Materials and Methods). ** *p* CURC-treated cells vs. control cells; * *p* < 0.05 CURC-treated cells vs. cells incubated in the absence of CURC; ^#^ *p* < 0.05 RT-treated cells vs. control cells (cells in medium without RT).

An impairment of proteasome function, which could reduce the cellular protein clearance, is considered as one of the mechanisms responsible for the accumulation of oxidized proteins in PC12 cells treated with RT. The literature data demonstrate that RT affects proteasome activity [31,32]. Thus, we evaluated proteasome activity in PC12 cells incubated with RT in the absence or presence of CURC (10 μM). As shown in Figure 4, CURC alone promotes proteasome activity, and the highest concentrations of RT significantly inhibit proteasome, when compared with controls. In lysates from cells co-incubated with RT and CURC, the proteasome activity was higher than in samples obtained from cells incubated with RT alone (Figure 4). This result indicates that CURC could reduce the accumulation of RT-induced oxidatively modified proteins by restoring, at least in part, the proteasome activity.

## 3. Discussion

The present study shows that CURC exerted a protective effect against oxidative stress by reducing both the ROS production and the levels of carbonylated and nitrotyrosine-derived proteins in RT-treated PC12 cells. In addition, we demonstrated that CURC is also able to protect cells against the inhibitory effect of RT on proteasome activity.

RT and other toxins/pesticides, such as paraquat and dieldrin, are widely used to develop in vitro and in vivo PD models [12]. Oxidative stress, among other mechanisms, is strongly implicated in RT-induced cytotoxicity and can potentially be targeted by antioxidant agents. Increased levels of oxidized proteins and alteration of the proteasome pathway are considered to be a cause of cytosolic proteinaceous aggregates, a hallmark of PD dopaminergic neurons [5,6,7]. CURC is a compound that has received considerable attention for its therapeutic properties, and it is considered to be a powerful antioxidant for its protective effect against oxidative and/or nitrosative stress in various cellular and animal models [24].

As previously reported [20], RT induced a dose-dependent decreased viability of PC12 cells and increased intracellular ROS levels; CURC reduced both cytotoxicity and FR raise induced by the toxin. These results are in agreement with previous studies carried out in other cell types treated with RT or with other toxins, such as paraquat and 1-methyl-4-phenylpyridinium ions (MPP^+^), also used to mimic Parkinsonism features [24]. In neuroblastoma SH-SY5Y [33] and SK-N-SH cells [34], RT induces oxidative stress that is suppressed by CURC derivatives. CURC treatment decreases the ROS production in SH-SY5Y and in PC12 cells transfected with α-synuclein [35,36]. The antioxidant effects of CURC are also demonstrated in in vivo PD models consisting of rats treated with RT [24,37].

Several studies using toxin-treated or transfected cellular models of PD demonstrated that CURC exerts its antioxidant effects by acting at different stages of oxidative-stress-induced pathways. Indeed, CURC contains a variety of functional groups that are probably all involved in its antioxidant activity. It can act by directly scavenging reactive FR and as a classical phenolic chain-breaking antioxidant [38]. Moreover, CURC increased the expression of antioxidant genes (i.e., *SOD* and *GPx*) [26] and the synthesis of antioxidant molecules, such as GSH [28,39]. These effects are probably mediated by the effect of CURC on some transcription factors. In fact, it has been demonstrated that this compound activates Nrf1/Nrf2 and inhibits NFkB translocation and IkB degradation, which, in turn, increase the expression of antioxidant enzymes [29].

Acting at different levels, CURC and its analogs protect against lipid and protein oxidation, reducing the levels of malondialdehyde, carbonylated and nitrotyrosine-modified proteins [24]. However, these effects seem to be depended on tissues, cells and experimental conditions (i.e., dose and incubation time) [24].

In our previous study, we demonstrated, and herein we confirmed, that RT induces protein carbonylation in PC12 cells [20]. Carbonyl group is considered to be a marker of protein oxidative damage, as well as one of the main hallmarks of oxidative-stress-related disorders such as neurodegenerative diseases [4,40]. In particular, increased protein carbonylation levels have been reported in various in vivo/in vitro PD models [16,17,41] and PD patients [42,43]. Notably, in the current study, we demonstrated that CURC reduced proteins’ carbonylation levels induced by RT in PC12 cells. No information is available, to date, regarding the effects of CURC on protein oxidation in dopaminergic cells exposed to RT. Results reported in this study are in agreement with data demonstrating that CURC is able to reduce carbonylated protein levels in Alzheimer’s disease and PD models [24,44]. Moreover, the protective effect of CURC rises with the increase of RT concentrations, suggesting that prooxidants condition could stimulate some biological properties of this polyphenol. Indeed, it has been postulated that some effects of CURC could be mediated by its nonenzymatic oxidation products that have been found in cell culture medium, as well as in vivo [45,46,47].

Nitration of tyrosine is considered to be the most harmful protein alteration induced by NO and NO derivative as peroxynitrite. Tyrosine is susceptible to nitration and oxidation, as it is often exposed at protein’s surface [48]. The nitrotyrosine-modified proteins have been described in PD *substantia nigra* and in Lewy bodies [49,50]. Their levels, in the clinical setting, have been proposed as functional and quantitative biomarkers of oxidative/nitrosative stress, correlated to the progression of neurodegenerative lesions [50]. In this study, we also evaluated the effect of RT alone or co-incubated with CURC on the level of nitrated proteins.

Our results indicate that RT exposure of PC12 cells induced the increase of nitrotyrosine-modified proteins in a dose-dependent manner. Although indirectly, these results suggest that RT is able to induce the RNS formation. In addition, our results indicate that this deleterious RT-induced effect could be reduced by co-exposure with CURC. It has been reported that CURC is able to protect cells against nitrosative stress, acting as a direct scavenger of peroxynitrite and/or activating antioxidant response through Nrf2 [51]. Protective effects of CURC on RT-induced NO production have also been observed in RT-induced cerebellar toxicity in mice [37]. Mythri and co-workers [51] demonstrated that CURC protects brain mitochondria against peroxynitrite effects, preventing 3-nitrotyrosine formation and increasing glutathione levels. No reports are available about effects of CURC on nitrosative stress in dopaminergic cells to date.

Unexpectedly, the levels of nitrotyrosine-modified proteins in PC12 cells exposed to CURC alone were higher, with respect to untreated cells. Nevertheless, Yu and co-workers [52] also demonstrated that CURC significantly increases the activity of neuronal NOS and the production of NO to levels compatible with protective effects in amygdala and hippocampus of aged mice [52]. Even if we are unable to explain the observed increase in nitrotyrosine-modified proteins induced by CURC alone, the protective effect on RT-induced nitration was still observed.

It is well-known that high levels of carbonyl- and nitrotyrosine-modified proteins induce the formation of proteasome-resistant aggregates [53]. Modest levels of carbonylated proteins are physiologically degraded by the proteasome system, while high levels of carbonylated proteins form high-molecular-weight aggregates that are not digested and accumulate in cytosol. These aggregates, which are a typical feature of neurodegenerative diseases [4], are resistant to degradation and can inhibit proteasome. Moreover, proteasome inhibition seems to increase neuronal vulnerability to normally subtoxic levels of FR [54]. Oxidative stress not only damages cellular proteins which are specifically targeted by 20S proteasome complex [55], but also oxidizes components of the degradative complex, compromising its functional integrity [56]. According to this, in a previous study, we demonstrated that most of the carbonylated proteins identified in RT-treated PC12 cells are components of UPS [20]. These results led us to hypothesize that these oxidative modifications could cause functional defects in the UPS degradative machinery [20].

In this study, we demonstrated that RT induces in PC12 cells a reduction of proteasome activity that correlated well with the increase of oxidized protein levels. These results are in agreement with previous studies demonstrating that RT inhibits the proteasome activity. Wang and co-workers [31] proved that pesticides, including RT, cause inhibition of proteasome activity in SK-N-MC neuroblastoma cells, overexpressing a GFP-conjugated proteasome degradation signal, through 20S and 26S complexes oxidation. More recently, Chen and co-workers [12] demonstrated that RT and other pesticides are able to induce mitochondrial dysfunction (i.e., mitochondrial fragmentation and ATP depletion), inhibiting 26S and 20S proteasome activity in SH-SY5Y cells. Moreover, in cultured neocortical neurons was observed a downregulation of transcription levels of all UPS players 15 h after the RT treatment, interpreted as one of the last stages in RT-induced cytotoxicity [57].

The pivotal role of the proteasome system in the degradation of oxidized proteins, whose intracellular accumulation represent one of the hallmarks of PD, is also suggested by in vivo studies. Indeed, many forms of familial PD are caused by mutations in UPS-component genes [58,59]. A decrease of proteasome activity has been described in *substantia nigra* of PD brains [60], while systemic exposure to proteasome inhibitors in rats caused motor dysfunction, the loss of dopaminergic neurons and the formation of inclusions resembling Lewy bodies [61].

Here, we demonstrated that CURC increases proteasome activity and counteracts the proteasome inhibition induced by RT in PC12 cells. This result suggests that one of the mechanisms engaged by CURC to reduce the levels of RT-induced oxidatively damaged proteins could be the activation of the proteasome. Moreover, CURC could restore the activity of proteasome by decreasing both the protein-oxidized levels and the oxidation of UPS components.

So far, no data from the literature report the effect of CURC on proteasome activity in PC12 cells. However, several studies have investigated the effects exerted by CURC on proteasome complexes in other cell types, with different results. Regulation of UPS machinery by CURC has been linked, among others, to transcription factors [62]. As previously reported, CURC activates the expression of Nrf1/Nrf2. Nrf1 is a promising target for therapeutic intervention in neurodegenerative diseases, due to its ability to enhance proteasome activity, whereas Nrf2 controls the expression of antioxidant and detoxification enzymes and thereby protects cells from the damage induced by oxidative stress [63]. The administration of CURC analogs in cell and animal models of spinal and bulbar muscular atrophy activates the expression of Nrf1 and Nrf2, which, in turn, increase the expression of downstream genes coding for proteasome subunits and other UPS components. Furthermore, it has been demonstrated that CURC was able to significantly ameliorate RT-induced dopaminergic neuronal oxidative damage in the *substantia nigra pars compacta* of rats, activating the Akt/Nrf2 signaling pathway [29]. Interestingly, the proteasome activity was upregulated by a low concentration of CURC and inhibited by a high concentration of CURC [62]. These different dose-dependent effects on proteasome activity might be, at least in part, explained by the evidence demonstrating that CURC could act as both a pro-oxidative and anti-oxidative molecule [64]. Moreover, differences in experimental conditions, such as CURC concentrations, duration of the treatments and especially cell types, may contribute to explain the variability observed on the effects of this polyphenol on the proteasome activity/expression.

## 4. Materials and Methods

### 4.1. Chemicals and Reagents

PC12 cells (rat pheochromocytoma cells) were obtained from Interlab Cell Line Collection, Genova, Italy. RPMI 1640 medium, horse serum, fetal bovine serum, penicillin and streptomycin were obtained from Invitrogen Life Technologies (Carlsbad, CA, USA). Dimethyl sulfoxide, rotenone, curcumin, 3-(4,5-di- methylthiazol-2-yl) -2,5-diphenyltetrazolium bromide (MTT), 2′,7′-dichlorodihydro fluorescein diacetate (DCFH-DA), trichloroacetic acid (TCA), 2,4-dinitrophenylhydrazine (DNPH) and Coomassie blue G250 were purchased from Sigma-Aldrich (St. Louis, MO, USA). Then, 2,4-dinitrophenol (DPN)-antibody was obtained from Molecular Probes (Eugene, OR, USA). Anti-Nitrotyrosine Antibody Merck Millipore (Billerica, Massachusetts, USA) Protein assay reagent, ECL system (Clarity-Western ECL Blotting Substrates) and polyvinylidene difluoride (PVDF) membranes were purchased from Bio-Rad (Hercules, CA, USA). The Proteasome Activity Fluorometric Assay Kit was purchased from BioVision Incorporated (Milpitas, CA, USA).

### 4.2. Cell Culture and Treatments

PC12 cells were grown in RPMI 1640 medium supplemented with 10% (v/v) horse serum, 5% (v/v) fetal bovine serum, 100 U/mL penicillin and 100 U/mL streptomycin. Cells were maintained in a humidified 5% CO_2_ atmosphere, at 37 °C, and passaged as needed. For experimental purpose, cells were seeded in poly-L-lysine-coated plastic culture plates, and, after 24 h adhesion, the culture medium was replaced with fresh medium containing increasing concentrations of RT (0.1–1 μM) or vehicle (0.05% DMSO) (Controls); then, cells were incubated for 24 h. For the evaluation of protective effects exerted by CURC, PC12 cells were co-incubated with different concentrations of RT (0.1–1 μM) in a culture media containing 10 μM CURC. This concentration of CURC was chosen on the basis of both cell viability experiments and previous investigations on PC12 cells [30]. The stock solution of CURC (2.7 mM) was prepared in DMSO.

### 4.3. Cell-Viability Evaluation

PC12 cells were seeded in 96-well culture plates (1 × 10^5^ cells/well), in 200 µL of culture medium. The following day, the medium was substituted with fresh medium containing RT and/or CURC. Following 24 h exposure, the medium was removed, and cells were treated for 3 h with MTT (1 mg/mL). Dark blue formazan crystals were solubilized in DMSO, and the corresponding absorbance at 570 nm was measured, using a microplate reader (Beckman Coulter DTX880, Beckman Coulter, Inc; CA, US). Cell viability was expressed as the MTT percentage reduction in treated cells compared with controls, assuming the absorbance of controls was 100% (absorbance of treated wells/absorbance of control wells) × 100.

### 4.4. Evaluation of Intracellular ROS Production

Dichloro-dihydro-fluorescein diacetate (DCFH-DA) is a non-fluorescent probe that passively diffuses through the cell membrane and is hydrolyzed by the activity of intracellular esterases, to form non-fluorescent DCFH, which is then rapidly oxidized to form highly fluorescent dichlorofluorescein (DCF) in the presence of ROS. PC12 cells were plated in 96-well black plates (1 × 10^5^ cells/well) and allowed to attach for 24 h, as described above. Cells were then treated with RT and/or CURC, and at the end of incubation, the culture medium was removed, and cells were washed with PBS and incubated with 10 μM DCFH-DA, at 37 °C, for 60 min. Then, cells were washed three times with PBS, and the fluorescence intensity of the oxidized DCF was measured at excitation/emission wavelengths of 485/530 nm, respectively, using a microplate reader (DTX880 Multimode Detector, Beckman Coulter, Inc; CA, US). Data (expressed as percentage of DCF fluorescence intensity with respect to control) were normalized to cell viability evaluated by MTT assay.

### 4.5. Oxidative-Protein-Damage Assessment

The main modifications occurring in proteins as a result of oxidative stress, including carbonylation reactions and peroxynitrite reaction with tyrosines to generate nitrotyrosine, were assessed by using specific antibodies by Western blotting.

Briefly, cells were treated with RT, and/or CURC were lysed in Ripa buffer (50 mM Tris–HCl pH7.4, 150 mM NaCl, 1% Triton X-100, 0.1% SDS, 0.5% Na-deoxycholate and proteases inhibitors) and centrifuged 10,000 g for 10 min. The supernatants were used to determine the relative levels for carbonylated, nitrated, after determination of total protein concentration by Bradford assay, using bovine serum albumin as standard.

In particular, carbonylated proteins were derivatized with DNPH by mixing 250 μg (1 mg/mL) of whole protein cell lysates with 50 μL of DNPH (10 mM) in HCl (2 N), vortexing and incubating for 60 min at room temperature. Proteins were precipitated with 300 μL of 20% TCA and centrifugated at 10.000 g for 10 min at 4 °C. The protein pellets were washed three times with 1:1 ethanol/ethylacetate, dried and then resuspended in Tris-HCl buffer pH 6.8 containing 0.2% SDS.

For Western blotting, 25 ug of proteins of dinitrophenyl (DNP)-proteins or cells lysate were separated by 10% SDS–PAGE and transferred to PVDF membrane. Membranes were incubated overnight at 4 °C, with the appropriate primary antibodies, anti-DNP (1:10000) or and anti-nitrotyrosine (1:1000), washed and then incubated with HPR-coupled anti-rabbit or anti-mouse secondary antibodies (1:5000). The immunoreactive protein bands were visualized by ECL and autoradiographic films. Film images were acquired by using a GS-800 imaging system scanner, and densitometry analysis was carried out, using QuantityOne 4.5.0 software (BioRad, Hercules, CA, USA). Relative levels of oxidized proteins, expressed as arbitrary units, were evaluated, normalizing the optical density (OD) of each whole lane on the films with the OD of the corresponding lane on the Coomassie-blue-stained PVDF membranes.

### 4.6. Proteasome Activity Assay

Proteasome Activity was determined by using a commercial Proteasome Activity Fluorometric Assay Kit. The assay is based on the chymotrypsin-like activity, utilizing a 7-amino-4-methyl coumarin (AMC)-tagged peptide substrate which releases free, highly fluorescent AMC in the presence of proteolytic activity. Briefly, after 24 h exposure with RT and/or CURC, cells were lysed with 0.5% NP-40, and measurement of proteasome activity was performed using the fluorometric assay in 96-well black-bottom plates, following the manufacturer’s instructions. The free AMC fluorescence was quantified at excitation/emission wavelengths of 380/460 nm, respectively, using a microplate reader (DTX880 Multimode Detector, Beckman Coulter). The relative activity was standardized by protein concentration, determined as described above.

### 4.7. Statistically Analysis

Data were expressed as mean ± SD of at least three independent experiments carried out at least in triplicate. Statistical comparisons were performed by using the one-way analysis of variance (ANOVA), followed by Tukey’s multiple comparison test. A *p* < 0.05 was deemed to be significant.

## 5. Conclusions

In this study, we demonstrated, for the first time, that RT not only induces protein carbonylation but also increases levels in nitrotyrosine-modified proteins in dopaminergic PC12 cells. Further, we showed that CURC is able to counteract some adverse effects of RT on this model of Parkinsonism. In agreement with previous studies, CURC reduced cytotoxicity and intracellular ROS levels. Moreover, CURC also reduced the levels of carbonylated and nitrotyrosine-modified proteins that resulted increased in RT-treated cells. Taking into account that CURC also restored the proteasome activity that was compromised in RT-treated cells, we could hypothesize that one of the protective effects of CURC, at least in PC12 cells, is exerted by the activation of the proteasome.

## Abbreviations

PD	Parkinson’s disease
RT	Rotenone
FR	Free radicals
RNS	Nitrogen reactive species
ROS	Reactive oxygen species
CURC	Curcumin
DNPH	Dinitrophenylhydrazine
PVDF	polyvinylidene difluoride
MTT	3-(4,5-di- methylthiazol-2-yl) -2,5-diphenyltetrazolium bromide
DCFH-DA	Dichloro-dihydro-fluorescein diacetate
UPS	Ubiquitin proteasome system
